# P22 A multifaceted staff-focused approach to improve urinary tract infection management in our Trust

**DOI:** 10.1093/jacamr/dlad077.026

**Published:** 2023-08-02

**Authors:** Faith Abraham, Hadeer Tawfik, Bala Subramanian

**Affiliations:** Doncaster and Bassetlaw Teaching Hospitals, UK; Doncaster and Bassetlaw Teaching Hospitals, UK; Doncaster and Bassetlaw Teaching Hospitals, UK

## Abstract

**Background:**

Urinary tract infection (UTI) is a common diagnostic label given to patients across all medical and surgical specialties. Despite this, there is a great degree of misconception on diagnostic and management aspects of this condition. Following our Trust performance in the NHS UTI CQUIN 2022/2023, where overall compliance was 41% and 48% for quarter 1 and 2, respectively, we embarked on a project to enhance our performance by identifying gaps in knowledge that resulted in over-diagnosis and inappropriate antibiotic treatment.

**Objectives:**

To optimize clinical diagnosis of UTI, reduce reliance on dipsticks in elderly and catheterized patients, and improve antibiotic stewardship.

**Methods:**

We surveyed 76 healthcare workers including doctors, nurses, advanced nurse practitioners and healthcare assistants to understand baseline knowledge and explore areas that required targeted intervention. Based on the survey results, we implemented an educational awareness campaign employing formal teaching sessions on specific wards, informal interactions, UTI flow charts and posters, new antibiotic guidelines and posts on the hospital communications and social media pages

**Results:**

From our survey, we identified 45% believed dipsticks were part of routine investigations, 47% performed dipsticks for catheterized patients and 79% believed that a positive dipstick in elderly patients indicated UTI. The CQUIN data involves five key parameters; including diagnosing UTIs and CA-UTI based on clinical signs and symptoms, establishing the diagnosis without reliance on dipsticks in elderly and catheterized patients, sending urine for culture and microscopy, changing or removing catheters where applicable and treating with an appropriate antibiotic. The individual compliance for each parameter was >60% but our overall compliance was poor. Our survey finding corroborated the initial CQUIN data in demonstrating that the major problem areas were dipstick use in elderly patients and catheterized patients and the use of inappropriate antibiotics for treatment. Following the first wave of our improvement strategies, we recorded a significant improvement in overall Trust CQUIN compliance of 51% and 58% for quarters 3 and 4, respectively, and on the wards where specific implementation strategies were carried out, an overall compliance score of 64%.

**Conclusions:**

The significant improvement we have made so far highlights what can be achieved with staff-focused interventions and the impact on patient care. We intend to continue to involve various specialities across the Trust to optimize UTI management.

